# Transitioning from Cyclosporine to Tralokinumab in Moderate-to-Severe Atopic Dermatitis: A Prospective Real-World Comparison of Direct Switch vs. Short Overlap

**DOI:** 10.3390/jpm15110515

**Published:** 2025-10-31

**Authors:** Clara Ureña-Paniego, Raquel Sanabria-de la Torre, Salvador Arias-Santiago, Trinidad Montero-Vílchez

**Affiliations:** 1 Dermatology Department, Hospital Universitario Virgen de las Nieves, 18012 Granada, Spain; clara.urena.sspa@juntadeandalucia.es (C.U.-P.); raquelsanabriadlt@gmail.com (R.S.-d.l.T.); or tmonterov@correo.ugr.es (T.M.-V.); 2 Department of Biochemistry and Molecular Biology III and Immunology, School of Medicine, University of Granada, 18071 Granada, Spain; 3 Instituto de Investigación Biosanitaria ibs GRANADA, 18012 Granada, Spain; 4 Dermatology Department, School of Medicine, University of Granada, 18071 Granada, Spain

**Keywords:** atopic dermatitis, tralokinumab, cyclosporine, biologic therapy, clinical dermatology, real-world evidence

## Abstract

**Background**: Cyclosporine (CSA) is a fast-acting systemic immunosuppressant frequently used in moderate-to-severe atopic dermatitis (AD), but its long-term use is limited by toxicity. AD affects as many as 20% of children and nearly 10% of adults worldwide and its chronic, recurrent course often requires several systemic treatment lines, making optimization of sequential therapy a high clinical priority. Tralokinumab, an IL-13–targeting monoclonal antibody, represents a safer alternative with a slower onset of action. This study aimed to compare the effectiveness and safety of tralokinumab initiated as monotherapy versus in overlap with CSA during the transition from conventional to biologic therapy. **Methods**: We conducted a prospective observational study involving 27 adults with moderate-to-severe AD treated with tralokinumab for at least 16 weeks. Patients were categorized into two groups: tralokinumab monotherapy plus topical agents (TM; *n* = 23) and tralokinumab initiated with a cyclosporine overlap for up to 12 weeks (TO; *n* = 4). Disease severity was evaluated using the Eczema Area and Severity Index (EASI), Investigator Global Assessment (IGA), and numerical rating scale (NRS) for pruritus at baseline and weeks 16, 24, and 52. **Results**: Both TM and TO groups demonstrated significant clinical improvement across all outcomes, with no statistically significant differences between groups (*p* > 0.05 for EASI, IGA, and NRS). At week 52, TM patients showed mean reductions of 18.66 (EASI), 2.21 (IGA), and 4.49 (NRS), while TO patients showed reductions of 15, 2, and 3.50, respectively. Tralokinumab was discontinued in eight patients (29.6%), most commonly due to lack of efficacy. Discontinuation rates did not differ significantly between groups. However, the very small size of the TO group (*n* = 4) substantially limits statistical power and any contrasts should be interpreted as exploratory. **Conclusions**: In this prospective real-world cohort, we observed improvement after initiating tralokinumab, with and without a short cyclosporine bridge. In light of CSA’s risks, TM may be considered a reasonable first-line systemic option. Prospective randomized studies are needed to determine whether overlap confers additional benefit.

## 1. Introduction

Atopic dermatitis (AD) is a prevalent chronic inflammatory skin disorder characterized by recurrent eczematous lesions and intense pruritus, affecting approximately 15–20% of children and up to 2–4% of adults in developed countries [1,2,3]. In Europe, adult prevalence ranges between 2% and 8%, with up to half of these patients experiencing moderate-to-severe disease [3]. Chronic or relapsing eczematous lesions, often accompanied by intense pruritus, sleep disturbance, impaired work productivity, and poor mental health, substantially reduce quality of life. Patients frequently report social embarrassment and stigma, and the itching can lead to disrupted sleep and cognitive impairment, further exacerbating psychological distress [1,2].

In moderate-to-severe forms, systemic immunosuppressants have traditionally been employed for disease control, with cyclosporine (CSA) being one of the most frequently used agents due to its rapid onset of action and robust anti-inflammatory effects. In fact, CSA remains the only conventional systemic immunosuppressant formally approved for the treatment of AD in patients older than 16 years, while other immunosuppressants are used off-label [3]. However, long-term use of CSA is constrained by its cumulative toxicity, particularly nephrotoxicity and hypertension, which can develop in approximately 10% of patients and require frequent monitoring. These safety concerns have catalyzed the search for alternative systemic treatments that offer durable disease control with a more favorable safety profile. The arrival of biologic agents has therefore triggered a paradigm shift from broad immunosuppression toward more selective immune modulation [4].

Tralokinumab, a fully human monoclonal antibody that specifically inhibits interleukin-13 (IL-13), has emerged as a promising therapeutic option. IL-13 plays a central role in AD pathogenesis, acting as a critical cytokine in the downstream cascade that results in skin barrier dysfunction and Th2 immune polarization, making it a logical therapeutic target for novel biologic therapies, and its blockade has demonstrated efficacy in reducing disease severity while preserving long-term tolerability. Preclinical studies indicate that IL-13 overexpression disrupts filaggrin processing and down-regulates key antimicrobial peptides, thereby exacerbating epidermal barrier impairment. Beyond direct cutaneous effects, IL-13 also modulates itch mediators and neuronal sensitivity, contributing to the vicious cycle of scratching and skin inflammation [5,6]. Unlike CSA, tralokinumab exhibits a more gradual onset of action, raising practical concerns when transitioning patients from fast-acting immunosuppressants [7]. In clinical settings, this transition phase poses a therapeutic dilemma. On one hand, continuing CSA in overlap with tralokinumab may help maintain disease control while the biologic reaches its full effect [3,8]. On the other hand, such a strategy prolongs exposure to CSA-related toxicities and complicates management due to the need for additional monitoring [4].

While some clinicians have adopted a bridging strategy—maintaining CSA for up to 12 weeks after initiating tralokinumab—others advocate for a direct switch to monotherapy, citing comparable outcomes in real-world settings. Despite growing clinical experience, the optimal length of such overlap remains largely empirical and varies widely among dermatology centers [3,9,10]. To date, no head-to-head clinical trials have directly compared these two approaches, and most guidelines derive their transition protocols from experience with dupilumab, whose pharmacodynamics differ from those of tralokinumab [7,11]. Extrapolating dosing and tapering strategies from one biologic to another may not capture the nuances of IL 13-selective blockade, underlining the need for specific evidence. Clarifying the optimal sequencing of therapies not only improves clinical outcomes but also informs healthcare resource allocation and patient counseling [3,4]. Taken together, these strategic considerations reinforce the imperative for clear, evidence-based guidance that balances efficacy, safety, and long-term healthcare sustainability.

Given the absence of standardized recommendations and the lack of comparative evidence between overlap and monotherapy strategies for tralokinumab initiation, further data are needed. The aim of the present study is to explore whether overlap regimens are justified by improved outcomes, or whether a direct switch represents an equally effective and safer alternative for patients with moderate-to-severe AD.

## 2. Materials and Methods

### 2.1. Study Design and Setting

We conducted a prospective, single-center, observational study in the Dermatology Department of Hospital Universitario Virgen de las Nieves (Granada, Spain) between January 2023 and February 2025. Consecutive adults with moderate-to-severe AD who started tralokinumab in routine practice were enrolled and followed for 52 weeks. Ethical approval was obtained from the local research ethics committee (protocol code HC01/0442-N-20), and this non-interventional prospective study was conducted under routine clinical practice conditions, and all data were collected in anonymized form. Informed consent was waived by the institutional ethics committee.

### 2.2. Participants

Inclusion criteria were as follows: (i) age ≥ 18 years; (ii) clinical diagnosis of AD according to the Hanifin–Rajka criteria [12]; (iii) baseline Eczema Area and Severity Index (EASI) ≥ 16 or Investigator Global Assessment (IGA) ≥ 3; and (iv) treatment with tralokinumab for a minimum of 16 weeks. Exclusion criteria were previous exposure to tralokinumab, coexistence of other inflammatory skin conditions that could confound assessment, use of other systemic immunomodulators, or withdrawal of informed consent. At screening, eligibility was confirmed and historical and clinical data (age at onset, prior systemic therapies or phototherapy, smoking status, prior atopic diseases and comorbidities), as well as baseline demographic information (age and sex) and comorbidities, were extracted from the electronic health records and recorded. Baseline laboratory screening included complete blood count, serum creatinine, transaminases, and a viral serologic panel. All patients received standardized training on proper biologic self-administration and adverse event recognition to promote adherence and maximize safety.

### 2.3. Treatment Strategies

Eligible patients were allocated, according to physician preference and shared decision making, to one of two initiation strategies:

- Tralokinumab monotherapy (TM)—subcutaneous tralokinumab 600 mg loading dose followed by 300 mg every 2 weeks, together with standard topical therapy.
- Tralokinumab with CSA overlap (TO)—the same tralokinumab regimen plus continuation of oral cyclosporine (2.5–5 mg/kg/day in two divided doses) for a maximum of 12 weeks post-tralokinumab initiation, after which CSA was discontinued. In this strategy, CSA was initiated de novo concurrently with tralokinumab at the index visit and used as a short bridging therapy with a predefined taper of up to 12 weeks. All patients had creatinine and blood pressure checked at weeks 16, 24, and 52 according to consensus guidelines.

No other systemic immunomodulators (e.g., corticosteroids, methotrexate) were permitted during follow-up, but stable topical therapy (emollients, topical corticosteroids, or calcineurin inhibitors) could be continued in both groups.

### 2.4. Clinical Assessments

Disease severity and symptomatic burden were prospectively evaluated at baseline (week 0) and at weeks 16, 24, and 52. End-points comprised the Eczema Area and Severity Index (EASI), the Investigator’s Global Assessment (IGA), and the 11-point numerical rating scale (NRS) for pruritus. Adverse events and treatment discontinuations (with reason and timing) were recorded at each visit.

### 2.5. Outcomes

The primary endpoint was the mean change in EASI from baseline to week 52 between TM and TO groups. Secondary endpoints included changes in IGA and pruritus NRS, as well as rates and reasons for tralokinumab discontinuation.

### 2.6. Statistical Analysis

Continuous variables are presented as mean ± standard deviation (SD) or median (inter-quartile range, IQR) and were compared with Student’s *t*-test or the Mann–Whitney U-test, as appropriate. Categorical variables were compared with χ^2^ or Fisher’s exact tests. Repeated-measures analysis of variance evaluated within-group changes over time. A two-sided *p* < 0.05 was considered statistically significant. Analyses were performed with JMP Pro version 16 (2021; SAS Institute, Cary, NC, USA).

### 2.7. Data Availability

De-identified data that support the findings of this study are available from the corresponding author upon reasonable request.

## 3. Results

### 3.1. Patient Disposition and Baseline Characteristics

**Figure 1:**
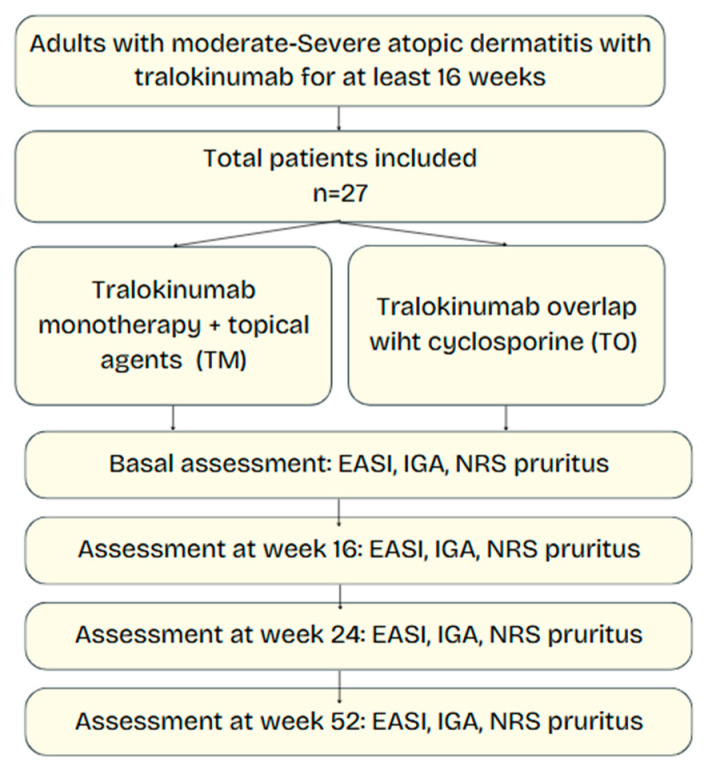
Flowchart of study design and patient allocation. Adults with moderate-to-severe atopic dermatitis were included in this prospective observational study and received tralokinumab for at least 16 weeks. Patients were divided into two groups: tralokinumab monotherapy (TM) and tralokinumab with overlapping cyclosporine for 12 weeks (TO). Disease severity was assessed at baseline and at weeks 16, 24, and 52 using EASI, IGA, and pruritus NRS.

Twenty-seven adults meeting the inclusion criteria were enrolled and completed baseline assessments. Figure 1 provides an overview of patient allocation. Tralokinumab was started as monotherapy in 23 patients (85.2%; TM group) and with CSA overlap in 4 patients (14.8%; TO group). Baseline demographic and disease severity indices were comparable; no statistically significant differences were found between groups (Table 1). Mean age was 39.9 ± 14.4 years in the TM group and 42.5 ± 22.5 years in the TO group; the majority of participants were male (52% TM, 50% TO). Baseline disease severity was comparable: mean EASI scores were 26.5 ± 9.4 (TM) and 23.0 ± 1.4 (TO); median IGA was 3 in both groups; and mean pruritus NRS scores were 7.8 ± 2.4 and 7.0 ± 1.6, respectively.

Mean age was 39.9 ± 14.4 years (TM) versus 42.5 ± 22.5 years (TO) (*p* = 0.79); baseline EASI was 26.5 ± 9.4 versus 23.0 ± 1.4 (*p* = 0.48); pruritus-NRS was 7.8 ± 2.4 versus 7.0 ± 1.6 (*p* = 0.46). Median IGA was 3 (IQR 3–4) in TM and 3 (IQR 3–3) in TO.

In the TO group (*n* = 4), median CSA daily dose was 200 mg/day (range 100–250; mean 187.5 ± 75.0). Median overlap duration was 11.2 weeks (range 8.6–12.0; mean 10.7 ± 1.6). Median cumulative exposure was 15,312.5 mg (range 6020–21,000; mean 14,411 ± 6587). Per-patient values are provided in Supplementary Table S1.

### 3.2. Clinical Efficacy

**Figure 2:**
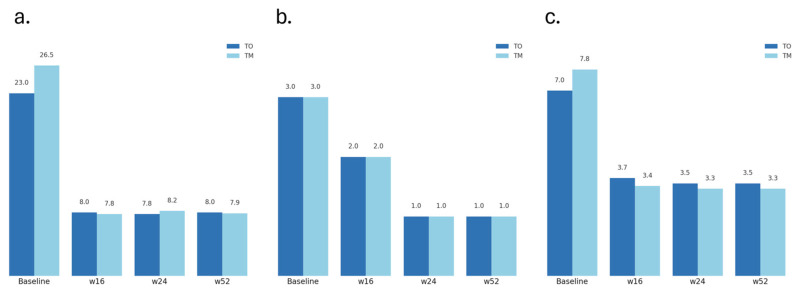
Mean EASI (**a**), IGA (**b**), and pruritus NRS (**c**) scores at baseline and weeks 16, 24, and 52 in patients receiving tralokinumab as monotherapy (TM) or with initial cyclosporine overlap (TO). Both groups showed comparable and sustained improvement throughout the study period, with no statistically significant differences at any measured time. EASI: Eczema Area and Severity Index; IGA: Investigator Global Assessment; NRS: pruritus numerical rating scale.

Both strategies produced rapid and sustained improvements in disease severity (Figure 2). In the TM group, mean EASI decreased from 26.5 ± 9.4 at baseline to 7.8 ± 6.1 at week 16, 8.2 ± 6.4 at week 24, and 7.9 ± 6.5 at week 52 (*p* < 0.001 for change over time). The TO group showed comparable improvements, with mean EASI decreasing from 23.0 ± 1.4 at baseline to 8.0 ± 5.7 at week 16, 7.8 ± 5.6 at week 24 and 8.0 ± 5.5 at week 52 (*p* < 0.01). When expressed as absolute change from baseline, the mean reduction in EASI at week 52 was 18.66 ± 9.8 for TM and 15.00 ± 6.6 for TO (*p* = 0.52).

At week 16, EASI50 was achieved by 10/23 (43%) TM patients and 1/4 (25%) TO patients; EASI75 and EASI90 were reached by 7/23 (30%) and 1/4 (25%) in each group, respectively. By week 52, EASI50, EASI75, and EASI90 were achieved by 5/23 (22%), 4/23 (17%), and 3/23 (13%) in the TM group, compared to 4/4 (100%) in all categories for TO. A total of 10/23 (43.5%) TM patients reached EASI50 by week 16 and 2 additional patients by week 52, while all TO patients (4/4) achieved EASI50—1/4 (25%) by week 16 and 3/4 (75%) by week 52.

Median IGA scores decreased from 3 (moderate) at baseline to 1 (almost clear) by week 24 in both groups. At week 52, 60.9% of TM patients and 50% of TO patients achieved an IGA of 0 or 1. Median IGA improved by –2.21 and –2.00 points, respectively (*p* = 0.47). No time-point showed significant efficacy differences between groups.

Pruritus improved substantially in both groups. Mean NRS decreased from 7.8 ± 2.4 to 3.4 ± 2.3 by week 16 and remained around 3.3 ± 2.5 at week 52 in the TM group, representing a mean reduction of 4.49 ± 2.7 points. TO participants reported mean pruritus NRS reductions from 7.0 ± 1.6 to 3.7 ± 2.4 at week 16 and 3.5 ± 2.2 at week 52 (mean change –3.50 ± 2.2). A ≥ 3-point improvement occurred in 73.9% (17/23) of TM and 50% (2/4) of TO participants. The difference between groups was not statistically significant (*p* = 0.49).

### 3.3. Treatment Discontinuation and Safety

Eight patients (29.6%) discontinued tralokinumab during the 52-week follow-up. Lack of efficacy accounted for six discontinuations (all in the TM group); two discontinuations (one per group) were due to mild adverse events (conjunctivitis and injection-site reaction). Mean time-to-discontinuation was 6.75 ± 4.31 months and did not differ between strategies (*p* = 0.34). No serious adverse events, infections requiring hospitalization, or clinically relevant laboratory abnormalities were recorded.

## 4. Discussion

Our study compared two transition strategies for tralokinumab in adults with moderate-to-severe AD: a direct switch to tralokinumab monotherapy (TM) and a strategy in which tralokinumab was initiated while overlapping with cyclosporine (TO) for up to 12 weeks before CSA discontinuation. Overall, both groups achieved significant and sustained improvements in major outcome measures such as EASI, IGA, and NRS; no between-group differences were observed within the precision afforded by our small TO subgroup.

Our results shows that adult patients with moderate-to-severe AD treated with tralokinumab—whether TM or TO—experience rapid and sustained clinical benefit. In quantitative terms, the mean reductions in EASI scores (–18.66 in the TM group vs. –15.00 in the TO group), IGA improvements (–2.21 versus –2.00), and reductions in pruritus NRS (–4.49 versus –3.50) at week 52 did not differ significantly between groups. These results are hypothesis-generating and suggest that the slower onset of action inherent to tralokinumab may not always require bridging with a fast-acting systemic immunosuppressant like CSA in order to maintain disease control. A similar conclusion is supported by prior multicenter prospective studies evaluating the effectiveness of tralokinumab in real-life settings, where comparable results were observed regardless of whether clinicians elected to use concurrent conventional immunosuppressants during the early treatment phase [3]. In addition, the overall rate of treatment discontinuation (29.6%) was similar between groups, and discontinuations were primarily due to lack of efficacy rather than safety concerns. This finding is consistent with data from earlier clinical investigations that reported low discontinuation rates for tralokinumab because of adverse events [13,14].

Historically, CSA has been the systemic treatment of choice in moderate-to-severe AD because of its rapid anti-inflammatory effects; however, its long-term use is marred by cumulative toxicity including nephrotoxicity, hypertension, and other systemic adverse events [14,15]. In contrast, tralokinumab is a targeted biologic that specifically blocks IL-13, a cytokine central to AD pathogenesis, thereby offering a favorable long-term safety profile [3,15]. Previous phase III studies (ECZTRA 1, ECZTRA 2, and ECZTRA 3) demonstrated that tralokinumab monotherapy could achieve statistically significant reductions in EASI and improvements in IGA and other quality-of-life measures compared to placebo [3,15,16]. In those trials, patients were managed with limited use of rescue medications, and the data indicated that long-term sustained disease control was achievable without concomitant prolonged exposure to systemic immunosuppressants. Real-world studies further underscore the effectiveness and tolerability of tralokinumab in clinical practice, with significant improvements in clinical outcome measures and a manageable adverse event profile [3,9,17]. In clinical practice, there has been concern that the delayed onset of action observed with tralokinumab may leave patients inadequately protected during the transition phase, prompting some clinicians to consider an overlap strategy with CSA. However, our findings indicate that TM results in long-term outcomes that are comparable to those achieved by the TO strategy. This suggests that the immunomodulatory effect of IL-13 blockade by tralokinumab is sufficient to attain sustained clinical improvement even without the auxiliary anti-inflammatory support provided by CSA during the early treatment period [3].

The novelty of our study is that it directly addresses the controversial clinical question of whether overlapping cyclosporine during the initial phase of tralokinumab therapy adds benefit. Various transition strategies have been adopted in clinical practice—some clinicians favor a short overlap to “bridge” the period during which tralokinumab’s slower onset of action might leave patients at risk of flare, while others opt for an immediate switch to avoid CSA’s toxicity [3,18]. Our findings are in line with recent observational data suggesting that a direct switch to tralokinumab monotherapy can yield clinical outcomes that are comparable to those achieved with a bridging strategy. Furthermore, indirect evidence from network meta-analyses and systematic reviews comparing various systemic treatments for AD indicates that biologics tend to provide consistent improvements in efficacy measures such as EASI and IGA scores independent of concomitant immunosuppressant use and our study lends further support to that notion [3,9]. Considered together, these data challenge the assumption that bridging is necessary and highlight the possibility that many patients may safely forego CSA once tralokinumab is initiated. Our findings should be contextualized within the broader framework of patient-centered care, where therapy choices align with individual preferences, comorbidities, and lifestyle considerations.

Tralokinumab’s role in AD management derives from its inhibition of IL-13 binding to IL-13 receptor, thereby attenuating downstream inflammatory signaling pathways implicated in skin barrier dysfunction and pruritus [15]. In contrast, CSA acts as a broad-spectrum immunosuppressant that interrupts T cell activation but lacks the selective cytokine targeting offered by tralokinumab, which theoretically could reduce the adverse events associated with generalized immunosuppression [14,15]. Our study’s finding that both initiation strategies confer similar clinical improvements suggests that the critical driver of long-term disease modification in these patients is the IL-13 blockade rather than the presence of temporary CSA-induced immunosuppression. This helps refine mechanistic understanding: once tralokinumab has effectively neutralized IL 13, the additional broad suppression of T cell activation may not confer further clinical benefit. Indeed, immunologic studies demonstrate that IL 13 blockade normalizes epidermal gene expression and reduces inflammatory cytokine levels, consistent with the clinical improvements observed [9].

The practical implications of our findings are significant when considering long-term management strategies in moderate-to-severe AD. Many AD patients are relatively young and require maintenance therapy for chronic disease control. Minimizing exposure to CSA could reduce the risk of long-term complications and lower the burden of frequent laboratory monitoring, thus facilitating a better quality of life and improved treatment adherence [3,4]. From a clinician’s standpoint, simplifying therapy by omitting CSA overlap also reduces administrative burden related to prescription renewals, drug interaction reviews, and cumulative dose calculations. Although biologics such as tralokinumab are associated with high upfront acquisition costs, the overall long-term economic burden of AD management may be lower when considering reduced monitoring requirements and lower rates of adverse events compared to conventional immunosuppressants [16].

Our study does have limitations that must be considered. First, our sample size was modest, and the TO group contained only four subjects. This small subgroup size may limit the generalizability of our findings and reduce statistical power to detect small differences between the two treatment strategies even when reflecting the reality of clinical practice. Second, this was a non-interventional, single-center observational cohort in which the choice between TM and TO reflected routine clinical judgment rather than random allocation. As a result, selection bias and confounding by indication—including unmeasured factors—cannot be excluded. Given the very small overlap group (*n* = 4), the study was not powered for between-group inference; accordingly, we deliberately refrained from covariate-adjusted or propensity-based analyses to avoid unstable, overfitted estimates and present unadjusted descriptive summaries only. Consequently, any apparent differences between strategies should be interpreted as exploratory and non-causal. Another limitation is that all included patients were biologic-naive at baseline, representing a somewhat selected population that might be more responsive to treatment than patients previously exposed to biologics. This could limit the generalizability of our findings to more treatment-resistant cases. Despite these limitations, our real-world observations support the pragmatic consideration of a direct switch to TM to limit CSA exposure, as we did not detect large or consistent differences versus short CSA overlap in this cohort, although confirmatory randomized studies are needed.

This information is crucial for clinicians as it provides a rationale for minimizing CSA exposure and its associated long-term risks while still achieving effective disease control in patients with moderate-to-severe AD. These results dovetail with the broader concept of treat-to-target in dermatology, whereby objective and patient-reported milestones are revisited at predefined intervals to tailor continuing therapy. Furthermore, our findings have economic implications: while the acquisition costs of biologics such as tralokinumab are higher compared to conventional systemic immunosuppressants, the overall cost may be offset by reduced monitoring expenses and fewer adverse events requiring additional medical interventions [3]. Randomized head-to-head studies comparing these two initiation strategies would provide more definitive evidence regarding efficacy, safety, and discontinuation outcomes.

Additionally, one additional area for future exploration involves the identification of biomarkers that may predict which patients are most likely to benefit from a direct switch to biologic monotherapy versus those who might require a short-term overlap with CSA. Although our study did not include biomarker analyses, ongoing research into the molecular underpinnings of AD suggests that certain serum cytokines and chemokines, such as TARC/CCL17 and PARC/CCL18, may serve as useful indicators of treatment response [19,20]. In addition, economic evaluations comparing the costs associated with CSA monitoring and potential adverse events to those of biologic therapy alone would also be valuable. Exploring sequential therapy paradigms—such as starting tralokinumab at a higher loading dose or combining with other targeted agents—may optimize early disease control without the need for broad spectrum immunosuppression. Understanding the heterogeneity in treatment response and tailoring transition strategies accordingly could further optimize patient outcomes and minimize unnecessary exposure to potentially toxic drugs. Personalized algorithms anchored in baseline biomarker profiles could, in the near future, dictate whether an individual truly benefits from CSA bridging.

## 5. Conclusions

In conclusion, in this prospective, real-world study of adults with moderate-to-severe AD, TM was associated with improvements in disease severity, pruritus, and global assessment scores comparable to those achieved with a short overlap of CSA. The findings should be interpreted as descriptive and hypothesis-generating rather than evidencing equivalence or non-inferiority but could nevertheless support the feasibility of a direct switch when minimizing CSA exposure is desired. Future confirmatory studies will be essential to further refine these findings and help integrate them into standardized clinical practice guidelines, ultimately offering a more optimized and patient-centered approach to AD management.

## Figures and Tables

**Table 1:** Baseline demographic, clinical severity, and comorbidity characteristics of the study population (overall and stratified by initiation strategy: global; TM; TO). No patient withdrew consent, and follow-up completeness exceeded 95%.

Variable 1	Global	TM (23/27)	TO (4/27)	*p*-Value
Age (years)	40.30 ± 15.31	39.91 ± 14.38	42.50 ± 22.49	0.837
Age at onset (years)	21.50 ± 25.90	20.30 ± 25.34	27.50 ± 38.89	0.838
Male (%)	16/27 (59.3%)	14/23 (60.9%)	2/4 (50.0%)	0.68
Asthma	6/27 (22.2%)	4/23 (17.4%)	2/4 (50.0%)	0.204
Food allergy	5/27 (18.5%)	3/23 (13.0%)	2/4 (50.0%)	0.14
Conjunctivitis	8/27 (29.6%)	6/23 (26.1%)	2/4 (50.0%)	0.56
Rhinitis	8/27 (29.6%)	6/23 (26.1%)	2/4 (50.0%)	0.56
Baseline EASI	26.00 ± 8.79	26.52 ± 9.44	23.0 ± 1.4	0.11
Baseline IGA	3.00 (3.00–3.50)	3.00 (3.00–4.00)	3.00 (3.00–3.00)	0.228
Baseline NRS	7.75 ± 2.34	7.82 ± 2.44	7 ± 1.6	0.54

Values are presented as mean ± standard deviation (SD) for approximately normally distributed continuous variables (age, age of onset, baseline EASI, baseline NRS); median (inter-quartile range, IQR) for skewed or ordinal variables (baseline IGA); and *n*/N (%) for categorical variables (male sex, asthma, food allergy, conjunctivitis, rhinitis). *p*-values were calculated with Welch’s *t*-test for continuous variables and two-sided Fisher’s exact test for categorical variables. TM = tralokinumab monotherapy; TO = tralokinumab combined with a short cyclosporine overlap; EASI = Eczema Area and Severity Index; IGA = Investigator Global Assessment; NRS = pruritus numerical rating scale; SD = standard deviation; IQR = inter-quartile range.

## Data Availability

Deidentified data supporting the findings of this study are available from the corresponding author upon reasonable request.

## Supplementary Materials

The following supporting information can be downloaded at https://www.mdpi.com/article/10.3390/jpm15110515/s1, Table S1: Cyclosporine overlap parameters in the TO cohort.

## Abbreviations

The following abbreviations are used in this manuscript:

AD	Atopic dermatitis
CSA	Cyclosporine A
EASI	Eczema Area and Severity Index
IGA	Investigator Global Assessment
IL	Interleukin
NRS	Numerical rating scale
TM	Tralokinumab monotherapy
TMO	Tralokinumab with cyclosporine overlap
