# Supplementary material for: Transitioning from Cyclosporine to Tralokinumab in Moderate-to-Severe Atopic Dermatitis: A Prospective Real-World Comparison of Direct Switch vs. Short Overlap
Source: J Pers Med. 2025 Oct 31;15(11):515. doi: 10.3390/jpm15110515 (PMC12653718; doi:10.3390/jpm15110515)
Supplement: Supplementary file 1 [file jpm-15-00515-s001.zip › jpm-3826341-supplementary.pdf]

Supplementary Material

Table S1. Cyclosporine overlap parameters in the TO cohort.

	Dosing	Duration (weeks)	Cumulative (mg)
Patient 1	250 mg/24 h	10.3	18,025
Patient 2	100 mg/24 h	8.6	6,020
Patient 3	250 mg/24 h	12.0	21,000
Patient 4	150 mg/24 h	12.0	12,600

Cumulative dose = (daily dose) × (weeks × 7 days).
